# Evaluation of Flour Safety Messages on Commercially Available Packages: An Eye-Tracking Study

**DOI:** 10.3390/foods11192997

**Published:** 2022-09-27

**Authors:** Merlyn S. Thomas, Zachary R. Berglund, Megan Low, Isabella M. Bryan, Reyhan Soewardjono, Yaohua Feng

**Affiliations:** Department of Food Science, Purdue University, West Lafayette, IN 47907, USA

**Keywords:** eye-tracking, flour consumers, food safety message, behavior change, perceptions

## Abstract

Wheat flour and baking mix have been associated with foodborne outbreaks and recalls, yet many consumers are unaware of the repercussions of consuming raw flour products. The objective of this study was to evaluate the accessibility of flour safety messages on commercially available packages and to identify consumer barriers to processing these messages. Eye-tracking technology was used to track the eye movements of 47 participants to assess their time to fixation (TTF) on the flour safety messages on 10 commercial packages. Notifications that were longer than one sentence were considered “long” messages, while notifications that consisted of only one sentence were considered short (S1–S5 and L1–L5). Only two participants (4.3%) found messages on all 10 packages. Highly accessible messages did not result in a high preference of presentation among participants. Most of the participants (98%) found the message on the S4 package, which correlated with the lowest TTF of 7.08 s. However, only 15% of those who found the S4 message chose it as their preferred message. Many participants who were interviewed said that they preferred messages that identified the reasoning for the warnings. They also preferred the messages that were well separated from other content on the package. Flour safety messages on the current packages are not effective to convey information and change consumer behavior. More science-based messaging strategies need to be developed to provide guidance for flour safety communication.

## 1. Introduction

Raw flour and other low-moisture foods are not traditionally considered a source of foodborne illness because their water activity (Aw) is less than 0.85 and bacteria growth does not usually occur below this point [1]. However, past outbreaks in these types of foods—such as peanut butter, rice, and wheat cereal—have caused foodborne illness in consumers [2]. Flour is a food of interest because it is a staple food ingredient in many consumer households and is used in many items, including other baking mixes and cookie doughs [3]. Even though it is a low- moisture food, there is a risk of microbial contamination because traditional milling of grains does not eliminate potential pathogens [4]. Consumers are, however, not aware of the risk involved in raw flour consumption. Many consumers throughout the ages have been eating or tasting raw doughs or batters [3,5]. A Food Safety and Nutrition Survey collected in 2019 showed that about 63% and 39% of participants did not think there was a likelihood of them getting sick from eating uncooked flour and raw homemade cookie dough, respectively; and 34% had tasted or eaten raw batter or dough in the previous 12 months [6]. There have been some reported cases of foodborne outbreaks as a result of flour consumption. From 2009 to 2019, six foodborne outbreaks in the U.S. and Canada were caused by wheat flour and flour products [7]. In 2019, a multi-state outbreak of *E. coli* in flour caused 21 cases and three hospitalizations [8]. An *E. coli* outbreak in 12 states was also linked to a cake mix in 2021 [9].

Consumers may not be aware of the repercussions of improper flour handling or even that any recalls have been related to it [3,10]. In a study by Feng and Archila, 85% of participating consumers reported having never heard of flour recalls or outbreaks [3]. In the same study, while 85% were confident in their flour handling, only 46% of those who cleaned their counters said they included a sanitizing step. If food contact surfaces are not properly cleaned and sanitized, potential pathogens can infect individuals via cross-contamination [11,12]. It is important to effectively encourage consumers to properly handle flour and to refrain from consuming it raw.

While many flour and baking mix packages contain warnings against consuming raw dough or batter, for various reasons those messages may not as effective as they could be. Only 22% of consumers participating in a previous study even paid attention to flour safety messages on packages [3]. Many factors can deter consumers from noticing or paying attention to these messages. Some of these may include the location on the packaging, the proximity of the message to other content on the package, the size of the message box and text, and color of the box and type. While the first step is to entice consumers to notice the message, the next step is to ensure that they understand the message and that it encourages them to practice proper behaviors. Previous studies indicate that the structure of the content (including stated benefits, reasonings, and other information) can greatly influence the effectiveness of a message [13,14]. Producers should communicate with consumers and document their comments carefully as part of a careful assessment of which factors can enhance consumer attention and retention of flour safety messages on packages.

Observations are an effective way to assess needs in real time. One way to track what consumers are looking at and how fast they can find messages is to record consumer eye movements using eye-tracking technology. Eye tracking is now often used in research to determine consumer perceptions, choices, willingness to purchase and their trust in food products by observing their interaction with extrinsic factors such as brands and packaging information [15,16,17,18,19]. Results from some eye tracking studies have shown a need for more in-depth research on characteristics of food packages that influence consumers attention as well as investigating labels on different types of food products [20]. Eye-tracking most commonly uses a technique that records the corneal reflection of infrared lighting to determine the pupil position. This allows the mapping of a person’s gaze and where they are focusing their attention [21,22]. This also allows researchers to collect metrics such as fixation count—how many times a person focuses on an area of interest (AOI)—and time to fixation (TTF)—how long it took to reach the AOI [22,23]. The TTF is useful for eliminating human error in determining how long a person requires to find the flour safety message on a food package. Eye tracking also can be used to assess what a person focuses on most often prior to finding the AOI and enables researchers to identify packaging elements that may be distracting consumers from noticing flour safety messages quickly. Distractions can include graphics or extra content, such as recipes and instructions.

The objectives of this study were (1) to utilize eye-tracking technology to accurately assess if consumers were able to find the messages on commercial flour and baking mix packages, and how long it took them to find the messages within a given timeframe; and (2) to assess attitudes and perceptions toward flour safety following the activity, to evaluate whether consumers would have a change in behavior intention.

## 2. Materials and Methods

**Figure 1:**
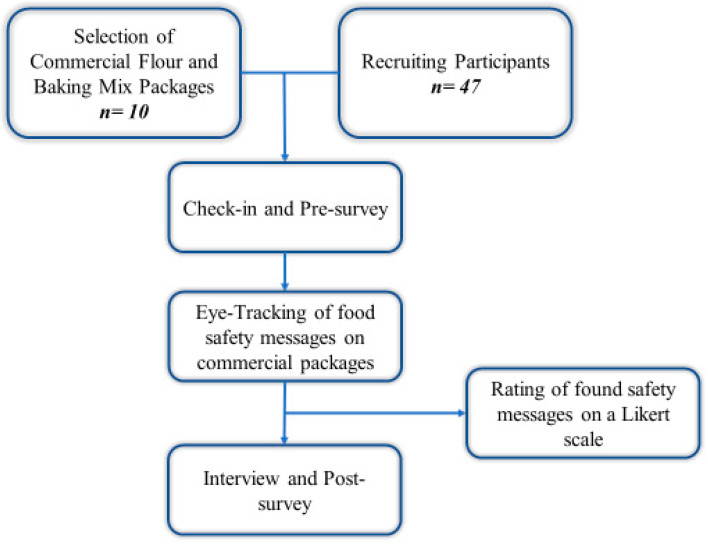
Data collection flowchart.

Study protocol was approved by the Institutional Review Board (IRB) at Purdue University IRB-2021-252 and pilot tested to improve techniques for data collection. The form of consent was signed by participants. The data collection process is presented in Figure 1.

### 2.1. Participant Recruitment and Check-In Procedure

Participants were recruited via a qualification survey using convenience sampling and snowball sampling methods. In the early stages of the participant recruitment, the Qualtrics XM consumer panel was used to reach qualifying individuals. Previous food safety studies have used this method to successfully recruit participants that fit certain quotas (age, gender, household role, etc.) [24,25,26]. In the case of this study, researchers were hoping to utilize the panel to randomly recruit flour consumers. However, due to the minimal responses, researchers decided to advertise the survey via flyers and snowball sampling to recruit additional participants. The qualification survey contained questions that would match the inclusion criteria for participating in the in-person study: primary food preparers or grocery shoppers for the household, use of wheat flour or wheat baking mix at least once a month, at least 18 years old, and ability to travel to the site of the study. Wheat flour included all-purpose flour, whole wheat flour, bread flour, cake flour, and other similar products. Baking mixes included cake mix, cookie mix, biscuit mix, pancake mix, muffin mix, brownie mix, and other similar products. Following recruitment, each participant was offered their choice of an hour-long time slot during which to complete the study. For completion of the whole study, participants each received an incentive of USD 75 to compensate them for their time and contributions.

After their arrival at the study site, participants were instructed to check in outside the study room. During check-in, they were given a consent form to sign and a pre-survey to fill out. The pre-survey assessed what type of wheat flour or baking mixes they used, if they sneaked a taste of raw flour products (cookie dough, cake batter, or bread mix), years of meal preparation experience, and demographic information (including gender, age, and ethnicity).

### 2.2. Eye-Tracking

The current study utilized eye-tracking technology to accurately assess the time to first fixation (TTF) or the time they required to visually find and recognize the area of interest (AOI) [23]. The AOI for this study was the flour safety message on the packaging. These messages contain warnings against eating or playing with raw dough or batter. The messages may contain other information, such as handling instructions and proper storage tips. Participants were asked to wear Tobii Pro Glasses 3 eye-tracker glasses (by Tobii Pro AB, Stockholm, Sweden). Analysis of eye-tracking data was done using the Tobii Pro Lab software (version 1.171.34906, by Tobii Pro AB, Stockholm, Sweden) to find the TTF for all packages and to create gaze plots for further analysis of one package. Gaze plots show spots on the package where the participant had a fixation and allow assessment of points at which they looked at first.

### 2.3. Commercially Available Packages

This study involved the use of 10 wheat flour and baking mix packages that researchers randomly selected from a chain grocery store (in West Lafayette, IN, USA). Prior to the random selection of packages, researchers had assessed all the flour and baking mix packages in the grocery store for food safety messages, and created a list of packages that contained food safety information. The random selection involved five packages with long messages (more than one sentence) and five packages with short messages (only one sentence). Two of the selected “long message” packages contained two separate messages. One of these two packages contained one short and one long message, but the package was still categorized as a “long message” package (Table S1).

The 10 packages were placed on the table in random order, and participants were allotted a maximum of 20 s to find the flour safety message on each package. Before participants moved on to another package, researchers re-started the timer, and the participants were told to focus on another point in the room during the transition in order to avoid looking at the next package prematurely. The time limit of 20 s was chosen based on previous eye-tracking research showing that consumers needed an average of 12.2 s to review a package and make a purchasing decision, as well as on another nutrition label eye-tracking study in which participants were given 20 s to view a food package [27,28]. Participants who did find the flour safety message were instructed to place that particular package to the side, and they were later given a set of statements that they had to rate on a Likert scale (1–7) based on their perceptions of each of the flour safety messages they found. The statements from which they had to choose were: (a) This flour safety message was easy to find, (b) this message was the perfect length for a flour safety message and, (c) in general, I prefer my flour safety message to be like this. To make sure that the participant had found the correct flour safety message, researchers asked participants to read the message aloud.

### 2.4. Interview and Post-Survey Questionnaires

After the eye-tracking activity, researchers conducted a brief interview containing questions about their experience with flour safety messages and their perceptions of those messages, their baking habits, and their use of ready-to-eat foods. Participants were also asked what other information they would like to see regarding flour safety messages. Following the interview, participants filled out a short post-survey questionnaire to wrap up the study. The post-survey form pertained to perceptions about flour safety messages and participants’ overall experience completing the study.

Post-survey responses were analyzed for descriptive data using Microsoft Excel (version 16.58, Microsoft, Redmond, WA, USA). The interviews were mainly analyzed using a deductive coding method for the responses from the questions asked, but also included some inductive coding for some noteworthy answers. The codes were further analyzed using a thematic approach [29]. The codebook was created by the main researcher analysis of the first 10 interviews using NVivo software (version 12.6.1, QSR International, Burlington, MA, USA) to organize the transcripts and codes. For further development of the codebook, another researcher independently coded three transcripts using the initial codebook and worked with the first researcher to reach consensus on the codebook (Table S2). Once the codebook was developed, the main researcher coded the remaining interviews and the same second researcher checked the contents under each code to make sure that the contents matched the definition of the codes.

### 2.5. Measuring Type Size

To investigate the influence of type size on accessibility of the flour safety messages on the packages, researchers measured the size of the type in millimeters. The body size is the distance from the tip of the highest ascender (top of the letter) to the lowest descender (bottom of the letter) of the tall letters [30]. In this study, the first letter was measured for each message. Our measurement excluded headings such as the phrases “Warning” or “Safe Handling Procedures”. One researcher measured the letters while another checked for accuracy. If packages contained more than one message, both messages were measured.

## 3. Results

### 3.1. Participant Demographics

Researchers recruited 47 participants to complete the study, and their demographic information can be found in Table 1. Most of the participants were non-Hispanic white (53%), and people identifying as Asian/Pacific Islander constituted the second-largest group (36%). Over half of the participants were 25–34 years old, females, or had a graduate degree. Many of the participants (66%) had over five years of meal preparation experience and lived by themselves or with one other person. Most of the participants bought all-purpose flour (94%) followed by baking mixes such as cookie mix, cake mix, and brownie mix (64%). Over half had tasted raw cookie dough (70%) and raw cake batter (53%) while baking (Table 1).

### 3.2. Accessibility of Flour Safety Messages on Commercial Flour and Baking Mix Packages

Table 2 displays the percentage of participants who were able to identify the flour safety message on packages. Corresponding package codes use the letter “S” to indicate packages with short messages and “L” to indicate packages with longer messages. Only two participants (4.3%) found all the messages on the 10 packages.

**Figure 2:**
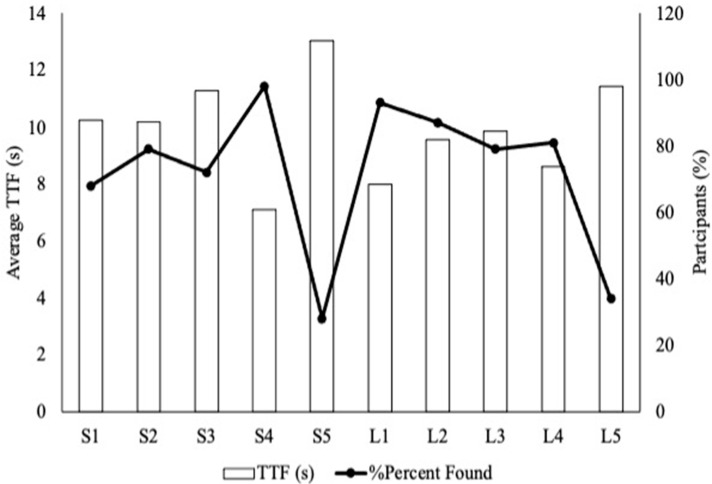
Overall average time to first fixation (TTF) for flour safety messages in Activity 1. Includes only participants who found the message in 20 s or less. The letter “S” (S1–S5) signifies that the messages were short and contained only one sentence. The letter “L” (L1–L5) signifies that the messages were long and contained more than one sentence.

While most people found the message on S4 (98%), only 15% of the participants preferred that message out of all the messages they found. S4 also had the lowest TTF of 7.08 s (Figure 2). On the other hand, S5 had the highest TTF of 13.04 s and the lowest percentage of identification (28%) (Figure 2). Along with that, none of the participants preferred this package. Due to the number of participants recruited for this exploratory study, statistical significance for eye tracking parameters could not be attained.

For the long messages, 93% of the participants found the message on L1, which attained the highest preference ranking (34%). L1 and L2 both contained two messages but L2 contained one short and one long. Researchers still classified L2 as long but when analyzing L2 separately, more participants (65.9%) found the long message (Figure S1). Like S5, L5 had the lowest identification among the long packages (34%), and only 13% of those who found it preferred it (Table 2). Participants also had the highest average TTF for L5 (11.42 s) among all the long packages.

**Figure 3:**
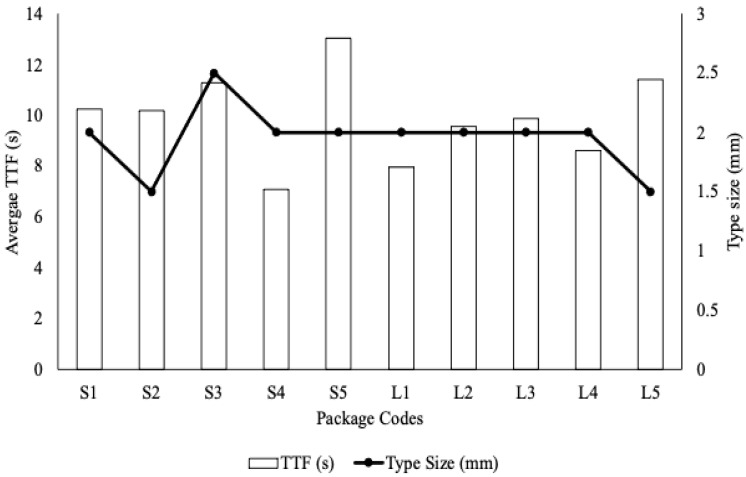
Overall TTF’s vs. type size for flour safety messages in Activity 1. Type measurements were taken from the top of the letters to the bottom of the letters and include only the message itself without any headings such as “Warning” or “Safe Handling Procedures”.

To consider if type size was a predictor of accessibility, researchers measured the first letter of the food safety message text on all the packages (Figure 3). However, the type sizes for most of the packages were similar (2 mm), and the package with the highest TTF (S5) was printed in this size as well. This may mean that other factors on the package, including color, contrast, position, and other distractions, may decrease the accessibility or conspicuousness of the food safety message.

Researchers further analyzed S5 recordings of those who found the food safety message, in order to assess what may have distracted the participant before they found it. Using the eye-tracking software, the researchers created gaze plots of the side of the package on which the message was printed. Table 3 displays the fixations of the 13 participants who found the flour safety message on the package. Most participants (5) first fixated on the “extra ingredients” table, which was located near the top of the package. None of the participants fixated first on the brand logo, the flour safety message or the website for tips. Right before finding the flour safety message, almost all the participants (10) fixated on the baking instructions near the middle of the package. Lastly, 10 participants had clusters of four or more fixations in some areas of the package. Most of them had clusters on the extra ingredients followed by yield and bake-time table (Table 3).

### 3.3. Interview and Post-Survey Responses: Capturing Participant Perceptions

After participants completed the exercise, they were each interviewed briefly and asked to fill out a post-survey instrument to gauge their thoughts and perceptions in response to their exposure to flour safety messages. Interviews were analyzed using a thematic approach; themes and subthemes can be seen in Table 4. Four subthemes and two themes emerged from this study.

All the participants except one thought flour safety messages were necessary for many reasons, including preventing foodborne illness among people who are not familiar with food safety, protection for the flour company, and correcting misinformation in news and social media. One participant commented: “Well, if I buy flour and I eat it not knowing that it could have bacterial contamination in it, and I get sick I could sue the flour company, and then of course, XXX (the company’s name) goes down like a ton of bricks—but they say, ‘sorry, we’ve got a warning on this and you did it anyway’, then that’s definitely going to protect the company” (female, 55–64). The one person who did not think the messages were necessary said it would be difficult for people to eat raw dough (e.g., pizza dough), and this person did not know that raw flour is in baking mixes as well.

Participants tended to prefer easy-to-find messages that were separated from other content and distractions on the package, as one participant indicated: “…the best message for me was one that was separated from too much text. I think it’s easier for consumers to find this safety message. … I saw some black boxes back there that the safety message was confusing to find because it was between too much text, so I think would be better for us to separate it from instructions or from ingredients…. [it’s] easier to find them [if] it’s clearer” (male, 18–24). Participants expressed divergent views about length preference. Some participants liked messages that were “short and to the point” (female, 45–54) while others liked information in longer messages explaining how and why someone could become ill. One person mentioned that the shorter messages may not contain enough information, thus leading consumers to believe that other factors (such as raw eggs) are the causes for consumers becoming ill. That participant wrote: “I think most of them [warning messages] are insufficient. The ones that we were reading, they were very short. They were hard to find on prepared mixes. I almost never notice it. And when I do, I don’t associate it with the mix—I associate it with the other things you’re adding to the mix. And so, I think [warning messages are] insufficient if you’re trying to really get that message that the flour being raw is what’s unsafe. That’s being lost” (female, 34–44). Along those lines, researchers noticed that when asked about future flour handling, some people mentioned they would store it in closed containers and in a cool place as some of the messages (L1 and L2) suggested (Table S1). They may be thinking that storage can get rid of bacteria. This participant said she would not change any of her flour handling practices because she stores it in cool temperatures: “No, because I always keep [it] in a cool temperature and then, you know, closed and [I] try to get rid of some insects. There’s no insects at home, but just in case” (female, 25–34).

Most participants had seen ready-to-eat labels and agreed that these types of foods should have very little to no preparation required. Participants expected that they would have to cook or prepare foods that are not ready-to-eat prior to consumption, in order to avoid becoming ill from bacteria. While this knowledge of not-ready-to-eat food was sufficient, most participants mentioned that they did not think flour posed a microbial threat. Reasons they cited for that belief included flour being made from plant material; its stability in shelf storage; it is considered an ingredient for cooking (not for consuming raw); wheat flour does not spoil; flour is a dry product; it’s not tasty or good to eat raw; and because they assumed that whenever they were told they should not eat raw batter or dough, it was because of the raw eggs and not the flour. Participants who did know they should not eat unprepared packaged foods mentioned they knew that because their relatives told them that eating raw dough would make them sick, or they had seen messages before, or were aware such food ingredients could cause indigestion, and/or they previously received food safety or agriculture-related education. One person was unsure if a particular ingredient was source of bacteria knew it could cause sickness said it’s: “not really a source of harmful bacteria that could make me sick, but I know that raw flour can make me sick … my grandmother told me” (female, 25–34).

Most participants said they had eaten or sneaked a taste of raw batter or dough before. Participants said they did so because they like to taste foods before baking them, or they had eaten raw eggs before, or they were vegan (and thus do not use eggs). One commented: “It’s fine if they don’t have eggs cause I’m vegan. I bake without eggs, and I eat raw cookie dough all the time” (female, 55–64). Again, consumers have been associating the harm of raw dough and batter coming from raw eggs rather than from the flour itself. When asked if they would continue to sneak a taste, most of the participants mentioned they would do so for various reasons, including that tasting the dough or batter has not caused illness or death yet, or the amount they taste is small, or everything has a risk, or because they have a strong immune system. One person who admitted tasting said: “Uh, yes, because I have never had any complications from it. I don’t tend to eat large amounts.… It’s in small quantities … typically shortly after making it. I guess you could say the freshest, it’s not been incubating for super long. Um, if there is that potential, especially cause it’s usually mixed with egg and there’s that Salmonella component. Um, so, I mean, I will, but it’s usually very infrequent. Um, so I will still probably do it” (male, 25–34). The few who indicated that they will not sneak a taste in the future said they would refrain from doing so because they already avoid eating raw dough or batter and/or they became aware after the study. Even so, some hesitation to refrain from eating raw dough apparently lingers because, as one participant said: “everyone does it [tastes raw batter] to check the batter” (female, 25–34).

Lastly, in response to being asked how they would handle flour in the future and overall, many participants said they would be careful. However, some mentioned they would not change their flour handling habits for the following reasons: they do not eat it anyway, they feel they handle their flour properly, and they have many years of experience. As mentioned earlier, some of the participants said they would just change their storage habits (storing in a cool place). However, some mentioned they will adopt behaviors like wiping down surfaces, washing their hands, and rinsing out their flour cup or other utensils they used. Others asserted that they would look for warning labels, and indicated they had become more aware of the repercussions, saying: “I’ll still be handling the same kind of way and make sure it’d be clean, clean up after myself and so on and so forth. So, I tend to be, and my wife as well, try to be thorough and making sure that everything’s baked fully, cooked fully, especially for our kids, to make sure that they don’t get sick. So, I won’t be doing anything different, but I probably might be looking out for those labels a little bit more” (male, 25–34).

The post-survey questionnaire responses also showed that most participants thought flour safety messages were important (98%) on food packaging, and one person thought it could “maybe” be important (Table 5). None of the participants reported that it would not be important at all. All the participants preferred the messages to be on the packaging itself followed by the address of the company website (30%) and the grocery store website (23%) (Table 5).

## 4. Discussion

### 4.1. Accessibility of Food Safety Messages

While having food safety messages on packaging is important, it may not be as effective if consumers are unable to find the messages within a reasonable amount of time. Out of the 47 participants, only two people were able to successfully find the flour safety message in 20 s or less on all 10 packages. In consumers’ daily food-related decision-making, 20 s can be a long time. A previous study indicates that consumers spend an average of only 12.2 s to view products before making a purchasing decision in a store environment [28]. Worse, consumers are not accustomed to look for safety information during that time, so it is critically important to ensure that food safety information is conspicuous and easily accessible.

Multiple factors may determine whether consumers are able to locate food safety messages. During the current study, researchers speculated that type size may influence the conspicuousness of food safety messages. When measuring the type size, however, researchers found no correlation between size and TTF. Conversely, a study done by Bialkova found that nutrition labeling caught consumer attention when the display size was doubled [31]. This indicates, for the current study, the size of type in safety messages on food packages may need to contrast more from the other text on the package in order to reduce the TTF value. Another proposed factor was color contrast or the color difference between the background and text that is overlaid on the background. Previously, researchers agreed that color contrast and the varied combinations of background colors and typefaces affect legibility [32,33,34]. According to the study by Bix et al., in which researchers measured the legibility of messages with differing color contrasts, black text on white background was the most legible combination for all age groups [32]. However, in the current study, S5 (the highest TTF) was also black print on a white background. This indicates the need for further investigation about what else could be impeding safety message accessibility and conspicuousness on certain packages.

Lastly, the researchers considered speculation that the placement of the message itself may affect accessibility. Aspects of placement include where a message appears on a package (front, back, top, bottom, side) and how distinct the message is from the text and graphics on the package. Participants in the current study even had mentioned in their interviews that they preferred messages to be in less crowded areas. Further analysis of S5 (the package with the highest TTF and lowest percentage of participants who found the safety message) revealed that consumers focused on many aspects of the package before finding the message. The other distracting elements include baking instructions, extra tables, and extra text. Similarly, a previous consumer eye-tracking study showed that health information was less retained by consumers than brand names and product names on food packages that contained a profuse amount of information [35]. In a previous analysis of over 100 flour and baking-mix packages, type sizes on most of the packages were similar to the type size used for the preparation instructions and ingredient list [3]. The packages selected for the current study were representative. Consumers were challenged to find flour safety messages among all the information on packages. While type size, color contrast, placement, and other information on the package individually may not be a factor in determining whether consumers are able to find food safety messages, combinations of those elements may reduce the accessibility and conspicuousness of food safety messages on packaging.

### 4.2. Information and Wording of Current Flour Safety Messages

Not only should these food safety messages be accessible, but they should also be easily understood. Scapin et al. wrote that information on food labels should be clear and easy-to-understand to help with consumer choices [36]. The book Thinking Fast and Slow, by psychology professor Daniel Kahneman, mentions that there are two systems for how a person thinks: system 1 and system 2 [37]. System 1 is automatic and requires very little effort because it comes naturally—such as understanding simple sentences or driving on an empty road—while system 2 allocates more attention and concentration to certain activities—such as looking for a white-haired woman or every letter a on this page [37]. Ideally, the flour safety messages on food packages should come easily (system 1) so that consumers understand these simple sentences.

Message preference is important to consider when creating food safety messages for consumers and to possibly evoke a system 1 thought process. A study by Hammond et al. showed that in communicating potential health risks of smoking, warning labels that were more comprehensive in content were more effective [38]. While consumers may be able to find a message, if they are dissuaded from reading it or have difficulty understanding it, the presentation of the message might not be effective in encouraging proper food safety behaviors. Overall, some consumers expressed no preference between short and long messages. A recent survey study assessing flour safety among consumers found that messages that include recommendations, explanations, and benefits of the recommendations were the most effective for preventing people from eating or playing with raw flour products [3]. Similarly, another study by Feng et al. discovered that the most effective messages for food irradiation contained information about the benefits of choosing the technology [11]. Another study also conveyed that information on warning labels that were more explicit were the most effective in gaining an emotional response from consumers [39]. While there is evidence to show that explanations and benefits are important aspects of an effective flour safety message, further research needs to be conducted to assess how much of this information is necessary without going overboard.

If messages are not clearly worded or optimally placed, consumers may be getting the wrong message or even misinformation. A paper by Swire-Thompson and Lazer define health misinformation as “information that is contrary to the epistemic consensus of the scientific community regarding a phenomenon” [40]. The wording of a message is important when it comes to conveying the proper information without accidental misinformation. For example, “store in a cool dry place” may cause people to equate that to “keep bacteria out” or “kill bacteria” rather than it being a way of quality control. This notion that storage may impact bacterial load was seen throughout the interviews in the current study. During the creation of messages, food safety experts and food companies need to be aware that accidental misinformation can be spread and may cause consumers to equate quality to safety.

### 4.3. Awareness Does Not Directly Affect Behavior Change

A consumer’s goals should be aligned with the health message in order to influence behavior change. A previous eye-tracking study related to nutritional labels found that consumers who had more nutritional goals spend more time attending to the nutritional information on food packaging [17]. Similarly, a more recent consumer flour study by Feng and Archila found that those who did not eat raw flour products found flour safety messages to be more effective than those who did consume these products [3]. Other studies have reported that the presence of front-of-package labels did not translate into healthier behaviors or choices [41,42]. In this current study, consumers expressed their thoughts through the interview and brief post-survey questionnaire. Overall, it seemed that most respondents were comfortable with their current flour handling and would not make many changes. Many even mentioned they would continue to taste or eat raw batter or dough because they did not ingest a large amount. This thought may have occurred because of optimism bias; they feel that they are unlikely to become ill because they have not become ill yet from eating raw flour products [43]. Another reason for this behavior may be subjective norms or the ways in which the actions of respondents are influenced by peers or people that they consider “important” [44]. One participant in the current study even asserted that everyone tastes raw batter to check it.

Another interesting point was that while these messages warned against eating raw dough or batter, a few participants attributed the potential for danger to the eggs rather than to the raw flour [3]. This indicates that consumers may need more education outside of flour safety messages on food packaging or extra information on the packages to clarify that both raw flour and eggs present the threat of danger. This is important because one participant mentioned that because she was vegan, she did not use raw eggs, so she recurrently eats raw cookie dough. This may be a common misconception among consumers. While flour safety education contributes to knowledge, the messages on the packages can act like a tool to increase perceived behavior control, since many consumers may use instructions on the package to guide their cooking.

This study has given researchers some insights about the accessibility and perceptions of consumers regarding flour safety messages and overall flour safety. More research needs to be conducted by observation and interviews in order to curate the most effective message content. Food safety experts and stakeholders must be aware of consumers’ perceptions regarding raw flour, and must appeal to them with a greater sense of urgency in order to initiate effective behavior change. Awareness and knowledge alone are insufficient unless packaging instructions provide consumers with accurate and complete information.

Although this study was carefully designed, some technical limitations need to be addressed. This study used eye-tracking technology to track consumers’ eye movements. Although the eye-tracker glasses and application were calibrated for each participant, some accuracy errors may have been introduced, however, due to technical difficulties involving the position of the glasses on the participant’s face, and the participant’s vision. In addition, while the selection of the flour and baking mix packages were done in a systematic way, the packages presented to survey participants may not necessarily have been representative of all the commercial packages available to consumers. Further research can be done to explore more packages and gather more data to support claims. Another limitation is this study was not conducted in a real- life setting, under regular purchasing situations. Despite this, findings from this exploratory study is valuable in guiding the development of flour safety messages. Further studies can be done to explore consumer behaviors in more realistic environments. Lastly, due to the COVID-19 pandemic and in efforts to keep researchers and participants safe, the study was done in only one community consisting mostly of people who attend or work at Purdue University. While this population provides rich data for this particular community and for further product safety labeling research, the participants may not represent all consumers. Again, additional and expanded research can be done in a way to encompass a wider participant pool. Authors should discuss the results and how they can be interpreted from the perspective of previous studies and of the working hypotheses. The findings and their implications should be discussed in the broadest context possible. Future research directions may also be highlighted.

## 5. Conclusions

This is the first study to examine the accessibility of flour safety messages on commercially available packages and to identify barriers that impede consumers from reading, comprehending and practicing the recommendations in these messages. Eye-tracking technology enabled measurements on consumers’ time to fixation (TTF) on the flour safety messages. The findings showed that participants encountered difficulty in finding flour safety messages on packages. Highly accessible messages did not result in high preference of presentation among participants. The preferred message was longer with both the recommendation of “not eating raw dough”, and the rationale of “flour being not ready-to-eat”. Factors that influence accessibility and conspicuousness need to be further studied. As a result of the limited number of packages used in this study, no clear correlation emerged between accessibility and the factors of font size, color contrast and placement of messages. Many participants who were interviewed said that they preferred messages that identified the reasoning for the warnings, and they favored safety messages that were well separated from other information on the package. This study sheds light on the use of eye-tracking technology to evaluate consumer accessibility and conspicuousness of food safety messages on packages. The findings will help guide the development of more effective flour safety messages for consumers and will support the decision-making process of flour industry stakeholders in consumer risk communication.

## Figures and Tables

**Table 1:** Demographic information of participants (*n* = 47).

Question	%(*n*)
**What type of flour or baking mix do you buy?**	
All-purpose flour	94(44)
Whole wheat flour	55(26)
Bread flour	40(19)
Baking mix (such as cookie mix, cake mix, biscuit mix, pancake mix, muffin mix, brownie mix)	64(30)
Other	11(5)
**Do you sneak a taste of the following items?**	
Raw cookie dough	70(33)
Raw cake batter	53(25)
Raw bread dough	30(14)
None of the above	23(11)
**What is your gender?**	
Male	32(15)
Female	68(32)
**What is your age?**	
18–24	19(9)
25–34	62(29)
35–44	9(4)
45–54	9(4)
55–64	2(1)
65 and above	0(0)
**What is your ethnicity? (Select all that apply)**	
White (non-Hispanic)	53(25)
Hispanic	2(1)
Black or African American	6(3)
Asian or Pacific Islander	36(17)
Native American	0(0)
Other	6(3)
**Would you give us a guess of your total household’s income (previous year) before taxes?**	
Less than USD 10,000	0(0)
USD 10,000–USD 29,999	51(24)
USD 30,000–USD 49,999	9(4)
USD 50,000–USD 79,999	13(6)
USD 80,000 and above	21(10)
Prefer not to answer	6(3)
**What is your education level?**	
Not high school graduate	0(0)
High school or GED degree	11(5)
Bachelor’s degree	28(13)
Graduate degree	62(29)
Prefer not to answer	0(0)
**How many years of experience do you have in preparing meals?**	
Less than 1 year	2(1)
1–3 years	9(4)
3–5 years	23(11)
Over 5 years	66(31)
**How many people (including yourself) live in the household?**	
1	34(16)
2	32(15)
3	13(6)
4	13(6)
5	9(4)
More than 5	0(0)
**Do you or the people living in the household have the following conditions? (Select all that apply)**	
Children younger than age 5	15(7)
People ages 65 and over	2(1)
Diabetes	0(0)
Immunocompromised, including organ transplant patients, HIV/AIDS, and cancer	2(1)
None of the above	83(39)

**Table 2:** Participants who found the flour safety messages in Activity 1.

Package Codes	Found Flour Safety Message %(*n*)	Those Who Preferred the Message %(*n*) ^b^
S1	68(32)	3(1)
S2	79(37)	14(5)
S3	72(34)	3(1)
S4	98(46)	15(7)
S5	28(13)	0(0)
L1 ^a^	93(44)	34(15)
L2 ^a^	87(41)	17(7)
L3	79(37)	16(6)
L4	81(38)	8(3)
L5	34(16)	13(2)

The letter “S” (S1–S5) signifies that the messages were short and contained only one sentence. The letter “L” (L1–L5) signifies that the messages were long and contained more than one sentence. ^a^ Package had more than 1 food safety message. ^b^ Only participants who found the flour safety message were asked this question.

**Table 3:** Gaze plot fixations for S5.

^a^ Area (from Top to Bottom of Package)	First Fixation, *n* = 13	Fixation before Flour Safety Message, *n* = 13	Clusters of 4 Fixations, *n* = 10 ^b^
Brand logo	0	0	0
Extra ingredients table	5	0	5
Baking instructions	3	10	5
Flour safety message	0	1	0
Yield and bake-time table	2	1	6
Extra recipe	3	1	7
Website for tips	0	0	0

Includes only participants who found the flour safety message on the package S5. ^a^ Displays the fixations only for the side of the package on which the flour safety message was printed. ^b^ Some participants had more than 1 cluster of 4. *n* represents the number of participants.

**Table 4:** Themes from interview codes.

Themes	Subthemes	Codes
Perceptions and preferences of flour safety messages	Flour safety messages were necessary	Compared to the grocery store
		General thoughts on flour safety messages
		Necessary messages
		Unnecessary messages
	Preference toward easy-to-find messages with mixed views on length.	Best message
		Additional information
		Other platforms for messages
Flour safety awareness increases but behavior may stay the same	Unaware of flour-related foodborne illness and continued consumption of raw batter or dough	Prior belief of flour safety
		Previous sneaking a taste
		Future sneaking a taste
		Future handling of flour
		Paying extra
	Knowledge and awareness of ready-to-eat products was sufficient among most with some exceptions	Ready-to-eat
		Ready-to-eat perception
		Not ready-to-eat perception

**Table 5:** Perception and preference of flour safety messages.

Question	%(*n*)
**Do you think it is important to have flour safety messages on packaging?**	
Yes	98(46)
No	0(0)
Maybe	2(1)
**How would you like to receive flour safety messages?**	
On the food packaging	100(47)
On the company website	30(14)
On the grocery store website	23(11)
Other	23(11)
None of the above	0(0)

## Data Availability

The data presented in this study are available on request from the corresponding author.

## Supplementary Materials

The following supporting information can be downloaded at: https://www.mdpi.com/article/10.3390/foods11192997/s1. Table S1: Food safety messages on grocery store flour and baking mix packages; Table S2: Interview codebook; Figure S1: Comparing the short and long messages on L2.
