# Supplementary material for: Evaluation of Flour Safety Messages on Commercially Available Packages: An Eye-Tracking Study
Source: Foods. 2022 Sep 27;11(19):2997. doi: 10.3390/foods11192997 (PMC9563750; doi:10.3390/foods11192997)
Supplement: Supplementary file 1 [file foods-11-02997-s001.zip › foods-1898178-supplementary.pdf]

Table S1: Food safety messages on grocery store flour and baking mix packages

	Package code	Message
Packages with short messages		
	S1	Please do not consume or eat raw cake batter
	S2	Do not eat raw cake batter
	S3	Do not eat raw cake batter or cookie dough
	S4	Do not eat raw batter, store in a cool, dry place
	S5	Do not eat raw batter
Packages with long messages		
	L1[a]	Say no to raw dough: flour is a raw ingredient. Bake fully before enjoying.<br><br>Do not eat raw flour, dough, or batter. Raw flour is not ready-to-eat and must be thoroughly cooked or baked before eating to prevent illness from bacteria in the flour. Do not eat or play with raw dough; wash hands, utensils, and surfaces after handling. After opening, keep cool and dry in a sealed container. Freeze for prolonged storage.
	L2[a]	Safe handling instructions: Raw flour is not ready-to-eat and must be thoroughly cooked before eating to prevent illness from bacteria in the flour. Do not eat or play with raw dough; wash hands, utensils, and surfaces after handling.<br><br>CAUTION: Do not eat raw flour, dough, or batter.
	L3	WARNING: Do not eat raw batter. Please cook fully before enjoying.
	L4	WARNING: Do not eat raw batter. Please cook fully before enjoying.
	L5	WARNING: Do not eat raw batter. Please cook fully before enjoying.

[a]Package had two separate flour safety messages

Table S2: Interview codebook

Code	Definition	Representative quote
1. Loss effectiveness	This code is when a participant receives a **loss** framed message in part 2 and talks about how effective it was as a flour safety message or how they felt about the message as a whole.	"Yeah, I think it would be effective. I do. I do understand this, well, shorter messages but I think for people that [are] not too related to the food safety area or food, like handling food, you will be able to message — put a little longer [message], like the effects it can have, and then one that says, you can become ill from the bacteria. I think that would be effective."
2. Gain effectiveness	This code is when a participant receives a **gain** framed message in part 2 and talks about how effective it was as a flour safety message or how they felt about the message as a whole.	"Yeah, I thought the message was kind of weird, because it's, like, enjoy baking if you do not consume, and it just seemed like the wording I was just not used to — I'm hearing usually it's do not consume, instead of if you do not. I don't know, it was just kind of weird to me."
3. Non-loss effectiveness	This code is when a participant receives a **non-loss** framed message in part 2 and talks about how effective it was as a flour safety message or how they felt about the message as a whole.	"I think yes, it will be an effective flour safety message…. Because it has all the messages, like properly cook before eating and why it is unsafe to eat without cooking it. And what could be the outcome. So, all three aspects of the warning are there."
4. Control effectiveness	This code is when a participant receives a **control** framed message in part 2 and talks about how effective it was as a flour safety message or how they felt about the message as a whole.	"Not really, because people still eat raw batter or raw cookie dough. I think [an] additional sentence might be helpful to say why you shouldn't."
5. Compared to grocery store	When a participant mentions what they feel is similar or different when it comes to the messages in part 2 (the messages researchers developed) versus the messages they see in the grocery store.	"I think it's not a big difference because these are actual products. The only thing at that, for this experiment, is time. I have enough time to look at the packages and inspect it and search for the place. But in the

			actual store I'm usually in hurry so I don't have the time to look."
6.	Ready-to-eat	When the participant mentions if they have heard of "ready-to-eat" or not.	"Ready to eat? Yeah."
7.	Ready-to-eat perception	When a participant mentions what they think ready-to-eat means. This can also be their expectations of the food itself.	"Foods ready to eat, I would assume that you don't have to cook it, or you don't have to wash it or anything like that."
8.	Not ready-to-eat perception	When a participant mentions that they think NOT ready-to-eat means. This can also be their expectations of the food itself.	"That it's raw, and you have to cook it or get it to a certain temperature, and you have to clean it too."
9.	Best message	When a participant mentions what they think is the best message for them, or if they mentioned a preferred way of seeing food safety messages.	"The best message for me was one that was separated from too much text. I think it's easier for consumers to find this safety message if it's separated from too much text. I saw some black boxes back there that the safety message was confusing to find because it was between too much text, so I think [it] would be better for us to separate it from instructions or from ingredients from all this message. For me what's easier to find is more clear."
10.	Prior belief of flour safety	When, prior to the study, the participant mentions their belief about flour being a source of bacteria.	"So actually, I've never considered flour as a source of bacteria, actually. Yeah, the way we use it in my country, for example, we use flour, raw flour, when we make tortillas or something like that. We do use some raw, so I don't think, for example, in my culture people are aware that raw flour can make you sick."
11.	Previously sneaking a taste	The answer that participants give when they are asked if they sneaked a taste of raw batter or dough before the study. This can be yes/no or an explanation/ experience they share.	"Yeah."

12. Future sneaking a taste	When participants mention whether they will sneak a taste of raw dough or batter after this study, and if they share an experience.	"Yeah, because I do know, now, the risk of trying raw dough and everything, and even though some people may find that it's good, you know, trying the raw dough, it's sweet and everything, but once you know the effect it can have on your health then you, yeah, I won't do it again." (He said "no.")
13. Future handling of flour	When a participant mentions how they will handle raw flour in the future.	"Yes, so I'll be more careful with handling because it's what you use, like some instruments in the kitchen that may have raw flour be more careful when using this; washing them — washing the utensils I use when I use the flours. Yeah, so being more careful and more detailed when using this."
14. General thoughts on flour safety messages	When participants express their general thoughts about flour safety messages on packaging.	"I do think they are necessary to warn people if you use it badly you can have an effect on your health. I generally think for me a shorter message aside from all the text that a normal text would have — it's more effective."
15. Necessary messages	When participants mention that the flour safety messages are necessary. They can also mention why they think this.	"Yeah, definitely necessary because many people, like maybe me before this study, did not know that you can [get] an illness from raw flour. Yeah, they're necessary for companies to protect themselves in case they have a case of any bacteria in any of their batches they can maybe protect themselves by putting the message on the package."
16. Not necessary messages	When participants mention that the flour safety messages are **NOT** necessary. They can also mention why they think this.	"Because I think it's very difficult that people eat raw dough. So probably this method can be the trend that people stop buying dough and buy directly the bread or the pizza dough without making it and probably it is worth

		it. When you're using the dough, you're usually going to bake it. So, all the bacteria are going to…."
17. Other platforms for messages	When participants mention other ways they would like to receive flour safety messages. They could say that the bag is fine or that there is no other way they would like to receive it.	"You think they can say a little bit more in their promotions, like when they promote their products on social media, or they can promote it a little bit more to make people more aware that they have to be more careful about using raw flour."
18. Additional information	When participants mention additional food safety information they would like to receive on the packages, in stores, on the website, or other places.	"Yeah, I think putting that consuming raw flour can make you ill is a general message for me that would be enough. Or maybe you can try to educate consumers to find that message because some people don't care, and only look for expiration dates or ingredients but do not look for the safety message — maybe educate consumers to find the safety message."
19. Paying extra	When a participant mentions whether they would pay more for pasteurized flour and why.	"Now that I know that it may have an effect on your health maybe yes. It depends on how much more, but, yeah."
20. Baking experience	When a participant shares more about their baking experience.	"Baking experiences, like when I go to buy flour, I never pay attention to the safety instruction, which I think I would do in the future. Uh, yeah, that's it, I think. I think this study, it was interesting. I didn't expect it to be."
21. Thoughts about the study	When a participant mentions their overall thoughts or takeaways from the study (last question).	I think it's good. I believe it's a good study to make people aware of these illnesses that raw flour can have. In my case, I never imagined that raw flour [could] make me sick, and it's because I don't know the perception we have. It's like it has low water activity and things like that, so

	it's really not a danger of having bacteria. But then this kind of study makes you realize that you can get really sick, so, yeah, I think it's a really good study for me and totally the rest of the consumers.

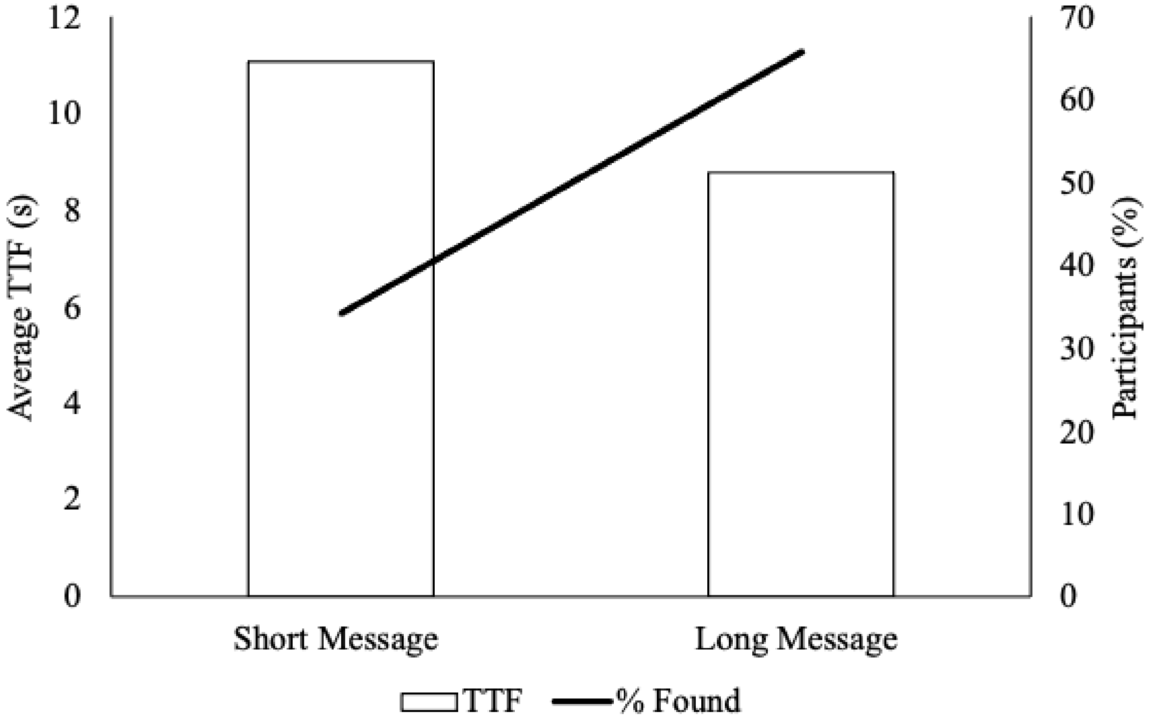

Figure S1. Comparing the short and long messages on L2
